# Australian policies on water management and climate change: are they supporting the sustainable development goals and improved health and well-being?

**DOI:** 10.1186/s12992-019-0509-3

**Published:** 2019-12-18

**Authors:** Toni Delany-Crowe, Dora Marinova, Matt Fisher, Michael McGreevy, Fran Baum

**Affiliations:** 1 Southgate Institute for Health, Society and Equity, Flinders University, Adelaide, Australia; 2 Sustainability Policy Institute, Curtin University, Adelaide, Australia

**Keywords:** Sustainable development goals, Planetary health, Natural environment, Social determinants of health, Well-being, Health equity, Climate change, Water

## Abstract

**Background:**

Sustainable management of the natural environment is essential. Continued environmental degradation will lead to worsened health outcomes in countries and across generations. The Sustainable Development Goals (SDGs) provide a framework for viewing the preservation of natural environments and the promotion of health, well-being and health equity as interconnected pursuits. Within the SDG framework the goals of promoting environmental sustainability and human health are unified through attention to the social determinants of health and health equity (SDH/HE). This paper presents findings from a document analysis of all Australian environment sector policies and selected legislation to examine whether and how current approaches support progress toward achieving SDG goals on water, climate change, and marine ecosystems (Goals 6, 13 and 14), and to consider implications for health and health equity.

**Results:**

Consideration of a broad range of SDH/HE was evident in the analysed documents. Related collaborations between environment and health sectors were identified, but the bulk of proposed actions on SDH/HE were initiated by the environment sector as part of its core business. Strengths of Australian policy in regard to SDGs 6, 13 and 14 are reflected in recognition of the effects of climate change, a strong cohesive approach to marine park protection, and recognition of the need to protect existing water and sanitation systems from future threats. However, climate change strategies focus predominately on resilience, adaptation and heat related health effects, rather than on more comprehensive mitigation policies. The findings emphasise the importance of strengthened cross-sectoral action to address both the drivers and effects of environmental degradation. A lack of policy coherence between jurisdictions was also evident in several areas, compounded by inadequate national guidance, where vague strategies and non-specific devolution of responsibilities are likely to compromise coordination and accountability.

**Conclusions:**

Evidence on planetary health recognises the interconnectedness of environmental and human health and, as such, suggests that ineffective management of climate change and water pose serious risks to both the natural environment and human well-being. To address these risks more effectively, and to achieve the SDGs, our findings indicate that cross-jurisdiction policy coherence and national coordination must be improved. In addition, more action to address global inequities is required, along with more comprehensive approaches to climate change mitigation.

## Background

Human well-being is dependent upon the health of the planet. Sustainable management of the natural environment is essential to support the health and health equity of individuals and communities, and is, therefore, a necessary focus for public health efforts.

Australia currently ranks highly in terms of environmental performance globally [1]. However the *Australia: State of the Environment 2016* reports emphasised that if current environmental trends continue, the Australian natural environment will have deteriorated significantly by 2050 [2]. The main pressures on the Australian environment are resulting from climate change, unsustainable land use and habitat degradation [2]. Australia is one of the world’s most resource and carbon intensive countries, contributing to depletion of finite resources and climate change [1]. The detrimental effects of climate change in Australia (including rising temperatures, fluctuating climates, and rainfall changes) are becoming increasingly evident, as is a reduction in groundwater quality [3]. Biodiversity across ecosystems is also poor and worsening [1, 3]. Recently Australia has experienced increased drought, a decline in the numbers of pollinators, and more regular extreme storms, which undermine food production [2]. Some crops are also becoming less nutritious due to atmospheric and soil changes [4]. Rising sea levels, ongoing demand for coastal land and severe weather events increase pressure on coasts [3, 5]. Pollution in Australian oceans is increasing, and climate change is warming and acidifying ocean waters, compromising marine ecosystem stability and diversity [6]. All of these trends also threaten human well-being, particularly by increasing the risk of natural disasters and detrimental climate changes, while potentially reducing access to clean air, safe drinking water and adequate food resources [2]. Such threats may lead to increased chronic and acute disease, particularly type 2 diabetes, obesity and overweight as well as cancers, heart disease and cardiovascular diseases, and reduced life expectancy [2]. With natural systems currently being degraded to an extent that is unprecedented in human history, both in Australia and around the world [7], there is an immediate need for action.

The United Nations’ *2030 Agenda for Sustainable Development* [8] views the preservation of natural environments and the promotion of human well-being and health equity as interconnected pursuits. The Agenda consists of 17 Sustainable Development Goals (SDGs) and 169 targets that countries across the world have committed to achieve by 2030. Achieving the goals requires countries to strike a balance in integrating social, economic and environmental pursuits. Recognition of human rights underpins the agenda, requiring partnerships between sectoral areas to ensure a standard of living for all that is adequate to sustain well-being. One of the SDGs, Goal 3, focuses explicitly on human health. However, all SDGs are interconnected, and almost all goals have some connection to the systems that influence human well-being and health equity- including governance of the natural environment [9, 10]. Recognising the interconnections between SDGs is imperative to mobilise resources for responsible environmental stewardship that will also protect human well-being [9, 11].

Our research critically examined Australian governments’ environment sector policies and the extent to which they do or do not address social determinants of health and health equity (SDH/HE). In this paper we draw on findings from the research to: a) examine whether and how the goals, objectives, strategies and values expressed in Australian environment sector policy and legislation documents will support action toward achieving three SDGs: 6, 13 and 14 (see Table 1); and b) consider the implications of such actions (or inaction) for health and health equity. The three SDGs have been selected because they are directly related to the core business of government environment departments. However, it is recognised in our research and in World Health Organisation documents on the SDGs, including the *Global Action Plan: Healthy Lives and Well-being for All 2019* [12], that environmental goals (and all goals) in the SDGs have clear links to the social determinants that influence human health, well-being and equity.

**Table 1:** Key focus areas of SDGs 6, 13 and 14

Sustainable Development Goals	Key focus areas for action
SDG 6: *Ensure availability and sustainable management of water and sanitation for all*	Fresh water resource management, safe drinking water, sanitation facilities, water scarcity, flooding, wastewater management and efficiency of water use.
SDG 13: *Take urgent action to combat climate change and its impacts*	Strengthen resilience and adaptive capacity to climate-related hazards, mitigate risks where possible, lower greenhouse gas emissions, improve education, awareness-raising and human and institutional capacity, and implement early warning systems.
SDG 14: *Conserve and sustainably use the oceans, seas and marine resources for sustainable development*	Sustainable use and conservation of oceans, overfishing, ocean acidification, expansion of protected areas for marine biodiversity, intensification of research capacity to preserve marine resources and reduce marine pollution.

Source: [8]

The analysis that is presented in this paper applies a planetary health perspective. Planetary health recognises that human health is inseparable from the broader environments and ecosystems in which individuals and communities live [7, 13]. This idea stems from the traditional knowledges that have been passed on within Indigenous cultures since time immemorial [14]. Planetary health views health not merely as absence of disease, but as a state of complete wellness at all levels- local, national and global [14]. In 2015 the Commission on Planetary Health offered the following definition:

*the achievement of the highest attainable standard of health, wellbeing, and equity worldwide through judicious attention to the human systems—political, economic, and social—that shape the future of humanity and the Earth’s natural systems that define the safe environmental limits within which humanity can flourish. Put simply, planetary health is the health of human civilisation and the state of the natural systems on which it depends.* [7, 1921].

This perspective situates human health within the context of the human made systems that are used to manage the environment. In doing so, it shifts environmental health risks, such as climate change and pollution, from being abstract forces to risks that can be understood, monitored, managed and mitigated by humans to realise positive change [7]. This dimension of planetary health justifies and encourages policy analysis as a research method because policy shapes how humans use and govern environmental resources. A planetary health perspective also asserts that humans live within a narrow “safe operating space of planetary existence” [7: 1921]. If the boundaries of that space are pushed too far, the conditions that currently sustain human well-being will be stretched beyond breaking point. This provides impetus for seeking policy change that will reduce risks to human health to fulfil an intergenerational responsibility to protect environmental systems, and allow future generations to thrive [7]. When it comes to protecting the natural environment, governments hold a lot of power and responsibility to act on behalf of the current and future generations. They exercise these through legislation but also through policies which guide action towards broader societal aims. This study analyses the policy landscape in Australia.

Interwoven throughout our policy analysis are references to neoliberalism. Miller and Orchard [15] discuss neoliberalism as an ideology that shapes public policy in Australia. Neoliberalism is based on the assumptions that sustained economic growth will have benefits that trickle down to benefit all people, and that free markets are most effective at achieving economic growth [15]. Neoliberalism also emphasises the importance of minimal state intervention in economic and social affairs [15], privileging individuals and businesses as entrepreneurial agents in generating capital and new markets. Miller and Orchard [15] argue that the neoliberal values that shape Australian policy often conflict with social democratic values, such as equity and social justice. Neoliberal politics in Australia has been characterised by the use of state institutions and resources to promote and/or protect private interests. Neoliberal ideas and values have also been used to justify the absence of strong policies on environmental protection by instead privileging narrow economic goals and pursuits. Where relevant, the concept of neoliberalism is drawn upon in this paper to illuminate some of the values that inform the policies, and that operate to shape the policy directions analysed in this paper.

## Methods

The research informing this paper examined how the policies of Australian governments in the natural environment sector influence population health, well-being and health equity [16]. It was based on a document analysis of strategic policy documents from environment departments in the state/territory governments and the national government of Australia (current at September 2016). Legislative Acts were also selected where they covered an area not addressed by strategic policy. Policies and legislation documents were selected for analysis because they guide the work of government decision makers as they determine which actions will be implemented and prioritised.

### Document sample

To identify the strategic policies and Acts, websites of relevant government departments in each Australian jurisdiction were searched between March and September 2016. A department was deemed relevant if it produced policy on any topic related to environmental protection or natural resource management. A list of relevant strategic policies and Acts was compiled from the website of each department. Documents were considered relevant if they were listed on the department websites at the time of the document search and appeared to still be active (i.e. not archived or superseded). Legislation was selected *only* if it covered areas of departmental policy that were not addressed by strategic policy from that department. The lists of policies and legislation were checked with public servants in each department to ensure currency and completeness. When additional strategic policies and legislation were suggested by public servants, these were added to the lists.

The final sample consisted of 178 strategic policies and Acts. All these documents were analysed using a coding framework that links the problem framing, values, goals, evidence, objectives and strategies stated within the documents with the various aspects of social determinants of health and health equity (SDH/HE) (see Table 2).

**Table 2:** Coding framework applied during the qualitative document analysis process

Focus area for coding	Coding categories	SDH/HE coded throughout all stages of the analysis
Problem framing	• What is the problem represented to be? • What response is considered appropriate by the government? • What else needs to be addressed? • How does the sector understand the relationships between their work and health and equity?	- Education - Food - Health systems - Housing - Distribution of income - Stigma/discrimination - Social relationships - Social exclusion - Transport - Employment - Welfare system - Land/Country (interdependent relationship between an individual and their ancestral lands and seas) - Gender - Safety - Culture - Open space - Natural environment - Built environment - Climate change [17–19]
Are the **values** stated in the document consistent/neutral/inconsistent with:	• health as a value • health equity as a value	
Are the **goals** stated in the document consistent/neutral/inconsistent with:	• improved health as a goal • improved health equity as a goal	
Is the **evidence** that is used in the document to make a case for action consistent/neutral/inconsistent with:	• evidence on social determinants of health • evidence on health inequities	
Are the **objectives** stated in the document consistent/neutral/inconsistent with outcomes to:	• improve average health • reduce health inequities	
Are the **strategies** stated in the document consistent/neutral/inconsistent with actions to:	• improve average health • reduce health inequities	

Source: Adapted from [14]

### Document analysis

Qualitative document analysis was employed to create a systematic procedure for reviewing and evaluating the structure, content and implications of the documents. Qualitative document analysis requires interpretation of data to elicit meaning and develop understanding about what is present and not present in the documents, and to what effect [20].

Each strategic policy and Act was entered into NVivo 11 and read at least twice. The first read was relatively superficial, aiding researcher familiarity. This was followed by a second reading which involved closer interpretation, with a focus on coding the content according to pre-defined categories (see Table 2). The framing of each strategic policy and Act was examined and the goals, objectives, strategies, and values articulated throughout were assessed to determine how and whether they aligned with the intent of progressing health, well-being and equity. Explicit mentions of words associated with health did not need to appear for segments of text to be coded. Instead, text *interpreted* as relevant to SDH/HE was also coded, guided by the list in Table 2.

The researchers identified silences within the strategic policies and Acts, where no aspect of the content could be coded into a particular category. Silences were also identified when the framing and goals of a particular document were inconsistent with its stated objectives and strategies, leaving some aspects relevant to health under-addressed or ignored within the proposed actions.

Peer-checking of the analysis occurred during regular team meetings between all authors, which involved collaborative re-coding and interpretation of policy segments. The implications of policy directions for population health, well-being and health equity were discussed during the meetings, as was the influence of ideology in shaping responses to environmental problems [21]. The final stage of the analysis involved checking the emerging findings against the SDG action areas to determine the extent of congruence, and identify areas where further action is required in Australian policy.

## Results

This section begins with an overview of the main topics and SDH/HE that were addressed within the analysed documents. This is followed by a more focussed presentation of key themes that emerged during the analysis relevant to SDGs 6, 13 and 14.

### Australian environment policies address a broad range of topics and social determinants

The analysed documents address an extensive list of topics (see Table 3) clearly related to SDH/HE and indirectly or directly related to SDGs 6, 13 and 14.

**Table 3:** Summary of main topics addressed by Australian environment sector policies

Topic	Details of foci in the environment sector policies
Population growth	Size of population, where the population lives and how people live
Climate change	As a risk to environment systems, economic productivity and human health
Air pollution	Particularly as associated with increased population growth and car dependency
Land use	Risks associated with land clearing, and potential environmental impacts of new infrastructure developments
Soil and water quality	Implications for food production, industry, and health of species
Water management and use	Pertaining to a range of aqueous environments such as rivers, lakes, wetlands and oceans. Management processes, human uses and associated risks
National parks and other protected areas, such as marine parks	Importance of controlled use to support conservation, biodiversity, and tourism. Economic opportunities associated with managed use of these areas
Renewable energy	As a green industry, with potential to reduce climate change impact and support transition in employment
Heritage and Crown land	Preservation of historically and culturally significant sites, rules to permit culturally significant activities in national parks, and co-management of land with Aboriginal and Torres Strait Islanders
Habitat destruction	As leading to species decline and/or extinction
Resources exploration and production	Impacts of exploration for oil, gas and coal, and the associated environmental risks
Environmental events and disasters	Particularly bushfire and storms (but also floods and droughts). Often linked to climate change
Waste	Production of excessive waste, ineffective handling, waterway pollution and potential for re-use

Multiple links to the SDH/HE were identified in the documents during discussion of these topics, with some links made explicitly and deliberately. Analysis of the goals, objectives and strategies that surround explicit discussion of health within the documents suggests that drawing links between environment sector business and the promotion of population health assists environment departments to establish the importance of their work within the broader agendas of governments. This was particularly apparent in Victoria and South Australia with strong emphasis on the co-benefits produced by ‘*Healthy Parks, Healthy People’* initiatives:

*Healthy Parks Healthy People is encapsulated in four key principles:*

*• the wellbeing of all societies depends on healthy ecosystems.*

*• parks nurture healthy ecosystems.*

*• contact with nature is essential for improving emotional, physical.*

*and spiritual health and wellbeing.*

*• parks are fundamental to economic growth.*

(Parks Victoria Shaping Our Future, page 4)

Climate change was also frequently linked to well-being both explicitly and implicitly, and to a potential decline in future well-being. In most instances an explicit focus on the well-being of vulnerable groups was evident:

*… climate change will have direct and indirect impacts on our health and wellbeing, particularly for vulnerable members of the community such as the elderly, those who live in remote settlements, the sick and people on low incomes. Health and community services will be affected across the state.* (SA Prospering in a Changing Climate, page 8).

Most often though relationships between the SDH/HE and environment sector business were implied rather than explicit. The SDH/HE coded most frequently in the data were: built environment, climate change, education, employment, land or Country connection, open space and transport. For example, discussion of employment was prominent in policies from all jurisdictions, especially in regard to the potential benefit of “*Supporting and promoting the employment of Aboriginal staff within natural resource management”* (NSW Fisheries Strategy and Implementation Plan, page 3). Fears about employment loss as a result of industry transition to cleaner fuels were directly countered by strategies to support increased employment in the renewable energy industry. Similarly, use of more ecologically sustainable waste technologies was advocated on the basis of employment generation:

*… there is often a lost employment and economic opportunity in disposing resources to landfill instead of reusing them. The Government will review the Tasmanian Waste and Resource Management Strategy to incorporate actions* (Embracing the climate challenge Tasmania’s climate change action plan, page 33).

During the analysis it became clear that countering such fears was one of the primary means through which the creators of the documents justified shifts in government policy away from practices that may support economic development, but damage the environment. Carefully balancing priorities appeared essential to garner support for new initiatives, because justifications based solely on the intrinsic value of environmental conservation may not have been sufficiently well aligned with governments’ neoliberalist economic agendas.

### Strong emphasis on preserving safe water and sanitation, but fragmented approach

Ensuring effective management of water and sanitation in all countries is imperative to realise SDG 6, and support health [22]. In general Australian drinking water and sanitation standards are high; however, the analysis identified several pressures on the sustainability of reliable, affordable and safe service provision. These include population growth, climate change and ageing infrastructure. Governance of drinking water and sanitation systems in Australia is also complicated by the diversity of systems that exist in different jurisdictions:

*Local government or local government businesses mostly provide drinking water and sewerage services, while the Queensland Government provides some bulk water supplies. While diverse in ownership, the sector also ranges in size and capacity. The largest service providers in South East Queensland service a population of more than one million people, while very small service providers serve some of the world’s most remote areas in the arid west and wet tropical north. Regardless of size and geography, every service provider needs to deliver services when, where and how they are needed by their local community.* (WaterQ: a 30-year strategy for Queensland’s water sector, page 7).

Under the Australian Constitution the states/territories have ultimate responsibility for managing water resources. However, the Commonwealth government also plays a role in developing national strategies because many of the pressures facing water and sanitation supply in each jurisdiction stem from nation-wide issues. Document analysis revealed that governments in all jurisdictions recognise that strengthened, coordinated action is required to ensure a clean sustainable drinking water supply. However, the findings also suggested that current systems are at odds with this because regulatory, governance and pricing mechanisms vary greatly across jurisdictions. Furthermore, the increasing privatisation of services has resulted in a loss of some direct control by governments, further complicating governance of water and sanitation, and management of their human-health impacts.

Much of the discussion in the analysed documents pertained to water and sanitation systems in urban areas. Less attention was directed to rural and remote locations where standards are often below that of urban areas due to poorer infrastructure, remoteness and more extreme climate conditions [23]. The documents remained almost completely silent on actions to address the greater expense and lack of reliability of services in very remote parts of Australia, which disproportionately affects Aboriginal and Torres Strait Australians, further reinforcing existing social and health inequities [24].

Drought, rising salinity and rainfall changes are future threats that were discussed in the documents as more likely in Australia as a result of climate change. These factors were identified in all jurisdictions as having the potential to compromise the future quality of Australian drinking water supplies, demanding innovative solutions:

*Extreme drought in the Murray-Darling Basin and the Mt Lofty Ranges has meant we can no longer use water as we have in the past – we need to be more efficient and much wiser when using our most precious resource … In future, our water supplies will feature climate-independent water through desalination. This ensures a portion of our water needs is guaranteed, despite increasing climate variability expected in future.* (SA Water for Good Plan, foreword).

While climate change is an ever-present threat to water systems, the extent to which effective and innovative action will eventuate to mitigate climate change is questionable. Thus, our analysis suggests that fragmented governance of water and lack of action on climate change are key risks to Australia’s capacity to contribute to SDG 6 and ensure universal access to safe water supply, which as water is a determinant of health will also undermine achievement of SDG 3 (Achieving good health and well-being).

### Climate change: considerable attention, but selective focus

In regard to SDG 13, all jurisdictions besides Queensland had policy/ies dedicated to action on climate change (*n* = 10). Furthermore, the need for action on climate change was expressed in nearly all documents (*n* = 124 of 178), even where the documents were primarily focussed on other topics. In the initial problem framing sections of the documents a broad array of climate change causes and impacts were identified:

*Our climate is warming at an unprecedented rate, largely as a result of human activity, and is already 1 °C higher than it was 100 years ago … The atmosphere and oceans have warmed, the amounts of snow and ice have diminished, and sea levels have risen. These changes are having, and will have, widespread impacts on human and natural systems.* (ACT Climate Change Adaptation Strategy, page 8).

Much of the explicit discussion about climate change-related health impacts was on the implications of rising temperatures and heatwaves, including more frequent heat-related illnesses and an increased prevalence of vector-borne diseases. Drought was also discussed frequently, with farming communities identified specifically as a vulnerable population. Less direct pathways between climate change and determinants of human health were mentioned only sporadically across the documents. Such pathways include climate change-related psychological stress contributing to mental ill-health and health risks from more frequent or severe weather-related events, including floods, bushfire, and storms [4]. The links between climate change and increased risks for social dislocation resulting from rising sea levels in Australia and neighbouring island countries were seldom discussed. Similarly the potential for climate change to contribute to food insecurity due to water insecurity and soil quality depletion was rarely mentioned [4]. Thus we found considerable attention to climate change in Australian policy, but generally inadequate examination of the complex relationships between climate change and social determinants of health.

In addition, the analysis indicated that far greater emphasis was placed on outlining climate change risks, rather than coherent, meaningful plans for action. To illustrate this trend, data on the topic of ocean acidification (relevant to SDG 14) will now be examined.

### Acidification of oceans: problem statements with few progressive mitigation strategies

Ocean acidification was identified as a concern in 17 of the documents. It was consistently linked to climate change, and explained as resulting from increased atmospheric concentrations of carbon dioxide. Acidification is problematic, particularly because it disrupts growth within marine ecosystems and impedes oceanic uptake of atmospheric carbon dioxide, potentially exacerbating climate change [25]. These environmental effects have major implications for food security and climate change as SDH/HE. Despite recognition of the risks associated with acidification, and understanding of the mechanisms by which it occurs, the inclusion of strategies for action was inconsistent, with few direct actions proposed in most of the 17 documents, and responsibility deferred to other areas of sectoral/government activity.

Where strategies for action were outlined, these were usually superficial and/or a continuation of actions that have already been implemented for a significant period, despite acidification continuing to increase since those actions commenced. An example comes from the Australian Government *Reef 2050 Long-Term Sustainability Plan*, which establishes clear relationships between acidification, climate change and environmental decline, suggesting dire consequences:

*The biggest long-term threat to coral reefs worldwide is climate change and the Great Barrier Reef is no exception. Damage to reefs as a consequence of climate change comes from ocean acidification, sea temperature increases, altered weather patterns (such as more intense storms) and rising sea levels.* (page 22).

*Future predictions indicate sea level rises and temperature increases will continue, the pH of the ocean will gradually decline and weather will be more severe.* (page 10).

The few strategies outlined to address ocean acidification in the Plan include repeated water sampling to provide “*Reef managers with information on where, when and how ocean acidification is affecting the Reef*” (page 61), and continued “*constructive participation in the United Nations Framework Convention on Climate Change (UNFCCC) and its Kyoto Protocol; through practical cooperation with regional partner countries; and through supporting developing countries to take actions that reduce emissions”* (page 61). The Government also committed to a modest objective of reducing emissions to “*five per cent below 2000 levels by the year 2020*” (page 61) (that is over a period of 5 years as the Plan was released in 2015). If that reduction were achieved it would represent a 19% decrease in emissions from the levels previously *projected* for 2020, rather than an *actual* decrease of 19%. As such, these strategies do not match the magnitude nor urgency of the problem of acidification that is conveyed in the problem framing statements, and their likely effectiveness in tackling further acidification, and health implications of this, is doubtful.

### Resilience rather than risk mitigation: emphasis on capacity to *respond* to climate change

Further exacerbating concerns about the likelihood of future effectiveness in tackling climate change is an overemphasis within the documents on achieving climate change *resilience*. This emphasis directs attention towards strategies such as vulnerability assessments, risk identification and management, and activities to adapt to changing climates:

*We will continue to build resilience to a changing climate within our natural environment and in relation to our Aboriginal and historical heritage values for future generations through:*

*• ongoing development and implementation of tools to support decision-making including assessing climate impacts;*

*• ongoing key research and monitoring programs; and.*

*• regulatory activity and collaboration with stakeholders.*

*As our understanding of actual and projected climate impacts increases we will adapt our approach accordingly.* (Embracing the climate challenge Tasmania’s climate change action plan, page 16).

While Queensland did not have a dedicated climate change policy at the time of data collection, a focus on achieving climate change resilience was replicated consistently throughout policies on other topics:

*This strategy will incorporate measures which contribute to the resilience of the Great Barrier Reef. In addition … A number of local governments are already preparing coastal hazard management plans and other initiatives in response to the anticipated effects of climate change.* (Reef 2050 Long-Term Sustainability Plan, page 23).

A focus on resilience can contribute to actions on SDH factors such as urban form or food security to moderate health impacts of climate change. However, our findings suggest that a focus on resilience is far more dominant than strategies to *mitigate* climate change. This is concerning as it may limit Australia governments’ capacity to contribute to SDG 13 and channel resources away from the more difficult – but essential – task of *preventing* the major, multiple risks to human health that climate change presents.

### Strong leadership on marine parks, but subordinance to mining interests

Consistent with SDG 14, Australia performs strongly in designating marine parks to provide for long term protection and conservation of particular ocean sites. Currently, marine parks exist in all Australian states and territories with coastal borders, covering 3.3 million square kilometres or 36% of Australia’s oceans [26]. Strict legislation was identified, with national and state/territory Acts that govern and restrict use of marine parks. All state/territory Acts indicate that they are coordinated through national legislation. This is demonstrated by a relatively cohesive approach to marine park management across Australia. Protection of marine parks can make a positive contribution to determinants of health and equity such as Indigenous people’s connection to country, the health of natural environments and food security.

Despite these strengths, analysis of the marine park Acts suggests another concerning theme: the subordinance of environmental conservation to commercial interests, particularly mining interests. A prime example is from the Western Australian Conservation and Land Management Act 1984 (page 8), which states:

*nothing in this Act shall derogate from the operation of the Mining Act 1978, the Offshore Minerals Act 2003, the Petroleum and Geothermal Energy Resources Act 1967, the Petroleum (Submerged Lands) Act 1982, (or) any other Act relating to minerals or petroleum*.

Such allowances have the potential to reduce Australia’s capacity to meet SDG 14 and to protect the contribution of marine parks to health.

### Marine ecosystem preservation: strong understanding of the problems but reactive actions

SDG 14 advocates sustainable use of oceans to manage and mitigate the threats associated with marine pollution, resource depletion and climate change. Action is essential to prevent further pressure on marine ecosystems and the health risks related to determinants such as compromised food security, loss of employment (e.g. tourist industry), and psychological stress from damage to revered ecosystems such as the Great Barrier Reef [27].

Our analysis reflects clear recognition of the wide variety of threats to marine environments, and the mechanisms causing these threats. Threats stem from increasing coastal development and pollution of coastal waters. The policies emphasised that pollution is driven particularly by fresh-water run-off from stormwater and agricultural lands. This run-off typically contains elevated levels of sediments, pollutants and nutrients, which may slow the growth of seagrasses and other species, while promoting the growth of invasive pest species. However, strategies within the documents focus primarily on reactive actions to counter problems after they have already eventuated.

One driver for the reactive approach is that there is little evidence in the policies of meaningful intersectoral strategising between environment and agricultural departments. Within the policies discussion of the agricultural sector focusses primarily on outlining how climate change will threaten future food production, and on monitoring environmental contaminant levels in food. Furthermore, the need to preserve the health and flow of rivers to service irrigated agriculture is a key driver for three Commonwealth Government policies. The apparent lack of collaboration to *change* detrimental agricultural practices, limits the power of environment departments to address the root causes of current problems, instead focusing attention on efforts to manage their effects.

Furthermore, it is acknowledged that continuing degradation of coastal ecosystems will exacerbate, and be exacerbated by, climate change. However, coastal protection strategies are largely administered separately from climate change action plans.

Thus there are several ways in which the potential for Australia environment sector polices to contribute to SDG 14 and protect the role of marine ecosystems in health appears to be limited by lack of coordination between relevant areas of policy.

### Balancing the human economic value of fisheries with ecosystem conservation

Preventing over-fishing is a key area of action in all jurisdictions and is fundamental to SDG 14, and also a significant factor affecting food security and health of natural environments as determinants of health. All 16 dedicated fishing related policies and Acts covered a range of issues (such as management of fish stock numbers in freshwater and ocean waterways, monitoring long term trends, development of local fisheries management plans, catch limits and fishing licenses). The documents reflected relatively consistent goals, emphasising a need to balance the interests of commercial and private fishers with ecological conservation and fish stock sustainability.

Despite some overall consistency, considerable differences were evident between jurisdictions, driven by the application of different values, about *which interests* should be prioritised. For example environment sector plans in the geographically small and landlocked Australian Capital Territory (ACT) referred to three primary water uses: *Conservation, Water Supply, and Drainage and Open Space*. Within each of these categories, a number of secondary uses were permitted provided that they were generally compatible with the primary uses. Such secondary uses included recreation (fishing, boating, swimming). Positioning fishing as a secondary use makes it less important than conservation of waterway ecosystems and biodiversity. It is important to note though because the ACT is landlocked its policies do not govern ocean fishing.

The strong emphasis on conservation in the ACT contrasts to varying extents with the value placed on preserving opportunities for fishing, economic gain and production in other jurisdictions. Although environmental sustainability is still a theme in these jurisdictions, the main focus is on sustaining environmental assets so that these can continue to be used. Fish stocks and associated ecosystems are valued primarily for the purposes of economic development, and to sustain the perceived “right” of individuals and commercial operators to fish for personal consumption or business pursuits:

*The Northern Territory’s fish stocks and aquatic habitats will be managed to maintain a quality recreational fishing experience for current and future generations.* (Northern Territory Recreational Fishing Development Plan 2012–2022, page 4).

Again, across the data set, few references to fishing practices, or strategies to control these, were included in climate change policies. This was surprising given the strong links between warming oceans, severe weather events, declines in fish numbers and degradation of waterways [28]. To contribute to SDG 14 and protect fisheries as important contributors to food security, Australian governments should prefer the approach of the ACT, to prioritise protection of the aquatic ecosystems that underpin sustainable fisheries.

### Sporadic leadership detracts from a comprehensive national approach

Our findings indicate that weak national leadership may stifle progress towards SDGs 6, 13 and 14 and weaken environment policy action on SDH/HE. The extent to which national policy documents provide meaningful and effective national leadership is unclear for three reasons.

1. The Australian Government is directly responsible for *few* actions within national environment policies. Furthermore, most often Australian Government responsibility is contained to very specific tasks that are unlikely to prompt broad scale change, or to demonstrate leadership, for example:
  *Audit of existing waste infrastructure and local capability in selected remote Indigenous communities as part of essential services audit under the National Indigenous Housing Partnership.* (National Waste Policy, page 15)
2. Responsibility for implementation in the national policies is largely devolved to state, territory and local governments. However, this generally occurs in a vague and non-specific way. For example, it is made clear that the national policies serve to “guide” or “advise” the work of other governments, or to suggest “what can we do”, rather than designating clear responsibility or accountability for action. Furthermore, in the implementation plans attached to the national policies, specific governments are usually not identified. Responsibility is most often devolved generically to “State and Territory Governments” or “All Governments”. Responsibility is also sometimes assigned to generic groups of non-government stakeholders as in the example below: 
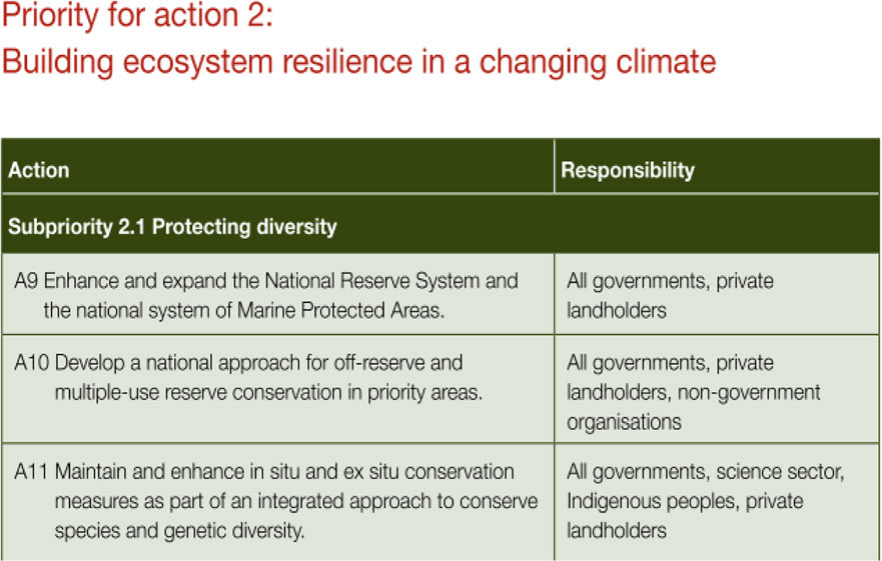
  (National Biodiversity Conservation Strategy, page 57)
  One exception to the generally generic attribution of responsibility is where specific natural resources are discussed, such as the Great Barrier Reef, and more specific jurisdictional designations of responsibility are made.
3. The wording used in the national policies is vague and left open to interpretation. Use of vague language may limit the power of the national strategies in stimulating meaningful action:
  development of **appropriate** management and, if required, disposal strategies where **appropriate** … *.*
  *Continued government* ***encouragement*** *of best practice waste management and resource recovery for construction and demolition projects.* (National Waste Policy, emphasis added)

### Doubtful policy translation to action due to relative lack of evaluation and monitoring

Overall, the researchers struggled to identify detailed, consistent plans for ongoing evaluation and monitoring of policy implementation across all policies in any of the jurisdictions. Most of the policies did not explain how progress would be monitored or whether there were budgets allocated to facilitate implementation. This is likely to impede the ability of Australian governments to accurately monitor progress in addressing environmental threats to health, and in achieving SDGs 6, 13 and 14.

## Discussion

Australian policy has several strengths in regard to progressing SDGs 6, 13 and 14 and, simultaneously, this means action is being taken to protect and promote human health by addressing social determinants of health. However, there are weaknesses that must be addressed. Through the lens of planetary health, current themes within Australian policy will now be examined under each of the three SDGs to consider the relevant environmental and human health implications, as well as some of the socio-political dimensions of current problems. As explained earlier, presenting a forward-looking approach to understand and shift the current nexus between environmental problems and human health impacts is a core component of a planetary health lens. As such, this discussion will consider strategies for policy improvement.

### SDG 6: ensure availability and sustainable management of water and sanitation for all

While Australia currently has relatively high quality drinking water and sanitation systems, the water related threats to human health presented by continued environmental degradation and worsened climate change are concerning. Such threats include restricted availability of adequate, clean river flows, food insecurity and potential increases in floods and the resulting social dislocation. A planetary health perspective stresses the importance of optimising governance systems [14]. The findings highlight a need across jurisdictions for a more cohesive, coordinated national approach to preserve water and sanitation standards and offset threats. Australian governments already invest in a coordinated approach, developed and administered through the Council of Australian Governments. Under the framework of the National Water Initiative, successive governments across Australia have also been working on a water reform agenda, which has the scope to improve coordination. To protect Australia’s water supply and sanitation as determinants of health it is imperative that this type of work continue, and be sustained over the *long term*, resisting the damaging impacts of short-sighted political visions and jurisdictional power battles that have impeded past reform efforts [29]. Furthermore, given that the research findings show continued fragmentation in water system management across the country, work must also be undertaken to examine *why* this is, and how broader adoption of consistent approaches can be facilitated to optimise use of the resources being invested in the national approach. Water reform work must also address the inequalities in secure water access that have emerged between rural and metropolitan systems to ensure that the SDG aspiration of water and sanitation *for all* can be realised [23], and that policies on water do not exacerbate existing health inequities between city and country regions in Australia. This is particularly important to support the well-being and health equity of Aboriginal and Torres Strait Islander people living in very remote country areas.

To build greater sustainability in Australia’s water supply, reform efforts must also acknowledge and challenge existing vested interests. A planetary health perspective encourages deep analysis of environmental management systems to consider how power manifests in ways that may compromise effective environmental management and human health improvements. The agriculture industry is a prime example of a powerful player. On average, agriculture uses 50–70% of the water consumed in Australia each year [30]. This is problematic, especially given that Australia’s water consumption per person is amongst the highest in the world, despite being the driest inhabited continent [31]. Large water withdrawals are damaging to rivers and thus likely over time to undermine the important roles that rivers have in supporting determinants of health such as food security, relationship to Country, and the health of natural environments.

Currently, the way that water is governed in Australia is not optimal. Siloed divisions of accountability and action direct responsibility for water system management to the environment sector while many of the levers for change remain unaddressed in other sectors. The relative silence on policy strategies to reduce the water consumption of the agricultural industry reflects siloed structures, but also the role of broader neoliberal government ideologies that privilege economic productivity in shaping policy responses. Many of the changes humans have made to freshwater rivers, such as diversions, and to land, such as clearing and deforestation, have occurred to support agricultural activities and other human resource production [31]. The majority of Australia’s surface water resources are also still used to service irrigated agriculture [31]. The amount of water used in agricultural production means that current, unsustainable production activities are likely to have much more impact than domestic water use. Furthermore, Australian cities only recently started to pay attention to adopting the principles of water sensitive urban design and have a long way to go before these aspirations become good practices [32]. This means that while the environment sector can impose water restrictions on private consumers and encourage water saving measures in the areas it controls, its power to conserve water remains limited. To achieve a truly sustainable approach to preserving Australia’s water resources, and protecting access to secure, affordable safe water as a determinants of health, it is, therefore, imperative that stronger synergies be achieved across environment, agricultural and urban planning policies [9].

### SDG 13: take urgent action to combat climate change and its impacts

Some understanding of the population health impacts of climate change on human well-being is evident in environment sector policies and so, to a degree, this reflects a planetary health perspective. Recognition of such relationships is an important step in progressing towards SDG 13. However there are areas of climate action where improvements are vital.

While Australian environment policies reflect understanding of the causes of climate change, the predominant focus remains on strategies to build community and system resilience to adapt to and accommodate these changes. It must be acknowledged that Australian mitigation strategies, in and of themselves, can have only a small impact on reducing the global greenhouse gas emissions that drive climate change. However, it is vital that all governments act to mitigate climate change, otherwise a tragedy of the commons will result. The tragedy of the commons is a situation in a shared-resources system, such as the global natural environment, where individual users act independently in pursuit of their own self-interests contrary to the common good to deplete the shared resources that all users depend on [33]. As such, a focus on resilience in Australian policy is important, but will be futile in protecting human well-being in Australia or globally without concurrent, strong action to mitigate climate change [34].

The findings of this study suggest that both the siloed organisation of policy and the strong economic focus of governments limit the power of the environment sector to deliver mitigation strategies. Given that decision making in all departments influences climate change, and that climate change is an increasingly influential determinant of human health globally [35], it does not make sense for action on climate change to be regarded only or primarily as environment sector business. Instead it should be positioned as whole-of-government business, with mandated and genuine collaboration across all sectors to halt environmental degradation and reduce greenhouse gas emissions [9]. Such a whole-of-government approach can be overseen by a central agency, with particular actions delegated to specific agencies across governments. This approach also offers the potential for more effective integration of a social determinants of health lens across policy areas.

Achieving a whole-of-government approach to climate change will necessitate clear and consistent articulation of the links between climate change and the broader agendas (particularly the economic imperatives) of neoliberal governments [34]. It will also involve utilising the collaborative intent of the SDG framework as a basis for building innovative and strong partnerships between governments, civil society and willing private sector partners, who can work together to use relevant technologies and build capacity [9, 36].

The public health sector of governments could assist by mobilising a planetary health agenda and re-orientating understandings of climate change as an ‘environmental issue’ to understanding of it as a whole-of society issue with major implications for human health [30]. This could be best done by offering effective, realistic and sustainable solutions for combating global warming, and supporting these with evidence and argument about the value of such measures for health and health equity. Focussing only on problems without also providing feasible strategies for effective change may be counterproductive by representing current environmental problems as bleak and unresolvable [34] and by underestimating the role Australia can play within this global agenda. Furthermore, the potential for whole-of-society benefit should be articulated, emphasising how human well-being and equity for all can be optimised through effective and well-coordinated mitigation action [9].

Another issue raised by the research is that currently climate change problem framings and resilience strategies direct disproportionate emphasis to populations living in vulnerable circumstances. Such emphasis is justified to an extent. People in vulnerable circumstances, such as low income earners and Indigenous peoples, are already more likely to experience ill-health and reduced life expectancy in Australia and around the world [4]. Climate change will worsen living conditions, and disproportionally affect those without good access to supportive systems and infrastructure, further compounding disadvantage [4]. Furthermore, there are groups for whom climate change effects will be particularly devastating; this includes farmers who suffer greatly during drought, flood and extreme temperatures as well as people living near the coast, whose homes may become inhabitable as a result of rising sea levels. However, the current emphasis on groups living in particular circumstances in policy encourages the perception that impacts will be largely restricted to these groups. This is not true as climate change will affect health and well-being across *all* population groups [34]. Therefore, those seeking to influence the debate about climate change and health must emphasise its *broad* impact, further appealing to the need for action from all sectors. Given the power of economics in influencing governance models, quantifying the likely costs of inaction in all sectors, and contrasting these costs with the enormous public health dividends of more effective reduction in greenhouse gas emissions and natural-resource management could offer an effective strategy [34].

### SDG 14: conserve and sustainably use the oceans, seas and marine resources for sustainable development

A strength of current Australian policy is ongoing commitment to the preservation of protected areas, including marine parks. Evidence on planetary health indicates that maintaining protected areas is essential to preserve biodiversity within marine ecosystems, increase fish numbers and support employment in strong nature based tourism industries [37]. However, outside of protected areas, current management approaches are concerning.

Policy and legislative control of fishing and ocean use in Australia is fragmented. Australia is already experiencing dwindling populations of some fish species that are caught for consumption and sale [6]. This is affecting the ecological balance of marine environments. On a global scale, similar problems exist, evidenced by a consistent decline in overall fish stocks and biodiversity during at least the past three decades [38]. This suggests that the predominant neoliberal values expressed in Australian ocean fishing policies, which prioritise economic interests and a perceived right to fishing over conservation [13], outside of designated marine parks are not supporting progress towards SDG 14, and may undermine sustainable fisheries contribution to food security as a determinant of health. Our findings suggest a need for stronger national leadership in this area, similar to that provided when marine parks were established, in order to offer strict controls over fisheries. Controls should aim to preserve existing stocks, and prevent further declines. A nationally coordinated approach to governing fishing would need to include multiple sectors, and to incorporate actions to protect natural resources from unfettered commercial interests. A more integrated approach is essential since fish populations are strongly influenced by the overall health of marine ecosystems, and these are currently threatened by various human activities apart from fishing [6, 26].

Fish should also be managed as part of the ecosystems in which they live, rather than being treated in isolation. An ecosystem-based approach goes beyond static catch quotas and involves continuous and responsive management to ensure that ecosystems can respond to climate change threats, and that all components of marine environments can be supported to flourish [28]. SDG 14 advocates for an expansion of research and monitoring to support conservation of marine environments. While regular monitoring is currently supported and resourced in many relevant areas of environmental management, such as monitoring of ocean acidification, this generally does not result in the monitoring of *all* components of an ecosystem in a way that recognises their intrinsic interconnections. Furthermore, resourced commitment to monitoring and evaluating the success of Australia’s own policy actions appears weak. More evaluation will show whether current policy action in Australia is proving effective in offsetting environmental risks before they translate into worsened human health, and create irreparable, widespread degradation of the environmental systems upon which human life relies.

A planetary health perspective also positions Australia’s responsibility to develop effective marine ecosystem management within a global context. The SDG framework highlights the interconnected nature of decisions made in countries around the world, emphasising shared accountability for positive change. Recognising the global health and health equity implications that will result from poor management of marine ecosystems and climate change mitigation highlights Australia’s obligation to address these issues effectively as a responsible global citizen. For example, food security as a determinants of health in many low-latitude low income nations is dependent on seafood [39]. Over the past three decades though, the worst declines in fish stocks have been in lower-latitude, low income nations [39]. Continued ocean warming is likely to drive remaining fish and shellfish species from low to high latitudes, potentially reducing fish catch even further in these regions, and globally by up to 30% by 2050 [25, 28]. Reductions in water oxygen levels and ocean warming mean that fish are also likely to get smaller in size, and coral reefs (essential for fish breeding and tourism) will be further degraded [28]. The implications of these trends are likely to hit low and middle income countries hardest [4]. Wealthy nations like Australia have compensated for declining fish stocks through intensive aquaculture production, by importing seafood from low and middle income nations at relatively low cost and by developing vitamin supplements [39]. Low income countries have fewer alternatives to make up for the shortfalls in population access to dietary nutrients, however, particularly as increased exports from these countries to wealthier nations are depleting fish stocks [39]. Imported fish and vitamin supplements are usually expensive, limiting access for those living in low income nations. As a result, communities are often forced to rely on what they can source locally and, increasingly, on less-nutritious processed foods [39]. We argue that high income countries, such as Australia, have an obligation to preserve their own local resources, to limit the export demand on low and middle income nations, and to take proactive action to reduce their impact on global marine ecosystems to prevent a worsening of global health inequities – now and in the future.

### Limitations of this study

This study conducted a census of all relevant policy documents and selected legislation to generate a comprehensive view of the environment policy landscape in all Australian jurisdictions. However, this approach has some limitations. To ensure a census of policy documents, data collection had to be bounded by set time periods. This meant that new documents could not be analysed as they were released or as policy directions changed. In addition, while the content of policy documents and legislation have a strong influence on the implementation activities of governments, these are not the only documents that influence government activity, and in some instances only parts of policies and legislation will be implemented by governments. As such, a more comprehensive understanding of government intent in the environment sector could be derived from also studying implementation activity and budget allocations. However, this was outside the scope of the current project. The authors also acknowledge that further insights into the topic of this paper could be gleaned from studying policies from all government sectors to determine what actions are being taken in all policy portfolios. The broader research from which this paper is drawn focussed on policies from four sectors (environment, justice, urban planning and energy), and also considered whole-of-government strategies (such as strategic plans) where these included goals related to the sectors of interest. However, government actions beyond this were not examined during the research. Furthermore, the design of the study did not allow direct evaluation of the population well-being impacts of policy interventions, instead links between the content of the documents and population health and equity were theorised by the researchers using existing literature and theoretical perspectives.

## Conclusion

The social determinants of health and the SDGs are intimately connected [12]. This paper has presented an analysis of the strengths and weaknesses of Australian environment sector policy in the pursuit of SDGs 6, 13 and 14, and in addressing social determinants of health to protect and promote human health equitably. We identified some strengths, including recognition of the importance of acting on climate change, the strict control of designated conservation areas and recognition of the need to preserve water and sanitation systems in the context of future threats.

However, a lack of comprehensive frameworks to address all drivers of climate change, and weaknesses in the management of waterways and marine ecosystems, still pose serious risks to the future of the natural environment and human well-being. While it is clear that promising policy directions are being pursued in several jurisdictions of Australia, current environmental risks are being compounded by weak national leadership. To address these risks more effectively, and to achieve the SDGs, our findings indicate that policy coherence across sectors and national coordination must be improved. In particular, a more comprehensive intersectoral approach to climate change mitigation is essential, which must acknowledge the interconnections between all elements of ecosystems, and be supported by cost-benefit analyses. An emphasis on the risks of climate change must also be accompanied by practical strategies for change, emphasising how climate change mitigation can improve quality of life for all.

Action to restore biodiversity and prevent further degradation will require major long term reinvestments to reverse environmental deterioration. This will necessitate political commitment to a vision of a sustainable and health-enhancing natural environment. Planetary ill-health is in part a product of the neoliberal ideology that dominates policy in Australia. This ideology creates policies that emphasise short-term economic interests, at the expense of protection for the natural environment. Shifting this focus and respecting the interconnectedness of human well-being and the natural environment is essential to progress the cohesive social agenda that underpins the SDGs, and, ultimately, will be vital to sustain human life on Planet Earth.

## Data Availability

Document lists and coding summaries are available from the authors upon request.
